# Identification of two putative reference genes from grapevine suitable for gene expression analysis in berry and related tissues derived from RNA-Seq data

**DOI:** 10.1186/1471-2164-14-878

**Published:** 2013-12-13

**Authors:** Mauricio González-Agüero, Miguel García-Rojas, Alex Di Genova, José Correa, Alejandro Maass, Ariel Orellana, Patricio Hinrichsen

**Affiliations:** 1 Instituto de Investigaciones Agropecuarias (INIA -Chile), La Platina Research Centre, Santiago, Chile. Av. Santa Rosa 11, 610, P.O. Box 439-3, Santiago, Chile; 2 Laboratory of Bioinformatics and Mathematics of the Genome, Center for Mathematical Modeling (UMI2807-CNRS) and FONDAP Center for Genome Regulation, Faculty of Mathematical and Physical Sciences, Avda. Blanco Encalada 2120, 6th Floor, University of Chile, Santiago, Chile; 3 Department of Mathematical Engineering, Center for Mathematical Modeling (UMI2807-CNRS) and FONDAP Center for Genome Regulation, Faculty of Mathematical and Physical Sciences, Avda. Blanco Encalada 2120, 7th Floor, University of Chile, Santiago, Chile; 4 Centro de Biotecnología Vegetal, Universidad Andrés Bello. Av. República 217, Santiago, Chile

## Abstract

**Background:**

Data normalization is a key step in gene expression analysis by qPCR. Endogenous control genes are used to estimate variations and experimental errors occurring during sample preparation and expression measurements. However, the transcription level of the most commonly used reference genes can vary considerably in samples obtained from different individuals, tissues, developmental stages and under variable physiological conditions, resulting in a misinterpretation of the performance of the target gene(s). This issue has been scarcely approached in woody species such as grapevine.

**Results:**

A statistical criterion was applied to select a sub-set of 19 candidate reference genes from a total of 242 non-differentially expressed (NDE) genes derived from a RNA-Seq experiment comprising ca. 500 million reads obtained from 14 table-grape genotypes sampled at four phenological stages. From the 19 candidate reference genes, *VvAIG1* (AvrRpt2-induced gene) and *VvTCPB* (T-complex 1 beta-like protein) were found to be the most stable ones after comparing the complete set of genotypes and phenological stages studied. This result was further validated by qPCR and geNorm analyses.

**Conclusions:**

Based on the evidence presented in this work, we propose to use the grapevine genes *VvAIG1* or *VvTCPB* or both as a reference tool to normalize RNA expression in qPCR assays or other quantitative method intended to measure gene expression in berries and other tissues of this fruit crop, sampled at different developmental stages and physiological conditions.

## Background

Quantitative real-time PCR (qPCR) is generally used for measuring transcripts abundance due to its high sensitivity, specificity and broad quantification range for high throughput and accurate expression profiling of selected genes [1]. Also, qPCR analysis has become the most common method for verification of microarrays and RNA-Seq results [2-4]. Besides being a powerful technique, qPCR has certain disadvantages such as the difficulties associated to the inappropriate data normalization, one of the most important aspects to solve [5] in order to fit this technique for the study of a new organism, organ or tissue. The data normalization is a key stage to control the artifacts and experimental error occurring during sample preparation and the following experimental steps, ending with the data analysis. It has been shown that qPCR results are highly dependent on the reference genes chosen [6], which explain the considerable effort applied into the validation of the gene(s) selected for the normalization stage, prior to extensive experimentation [7]. These housekeeping genes should not vary in their expression level considering the different tissues or cells under investigation, nor in response to any experimental treatment [8].

Regardless of the experimental technique employed, appropriate normalization is essential for obtaining accurate and reliable quantifications of gene expression levels, especially when measuring small expression differences or when working with tissues of different histological origin [9]. The purpose of normalization is to correct variability associated with the various steps of the experimental procedure, such as differences in initial sample amount, RNA extraction recovery and integrity, efficiency on cDNA synthesis and differences in the overall transcriptional activity of the tissues or cells analyzed [10]. Among the numerous normalization approaches that have been proposed [11,12] the use of internal controls or reference genes has become the method of preference [13,14], because they potentially account for all of the sources of variability mentioned above. However, numerous studies have reported that the transcript quantity of the most commonly used reference genes can vary considerably under different developmental, physiological and experimental conditions [11,15-23]. Several reference genes are commonly used, such as elongation factor [24,25], actin [26,27], ubiquitin [28,29], and ribosomal units (18S or 28S rRNA) [30-32]. However, several reports have demonstrated that transcript levels of these genes also vary considerably under different experimental conditions and consequently their suitability for gene expression studies must be evaluated case by case [22,33,34]. This implies that a reference gene with stable expression in one organism may not be suitable for normalization of gene expression in another organism [35,36], or even in different experiments for the same species.

Many works have been carried out on animal models and in relation to human health [37,38], fields in which multiple reference genes for normalization of qPCR data have been described. However, similar reports are less abundant in plants [10,35,39]. Czechowski et al. [22] employed a new strategy for the identification of reference genes in *Arabidopsis thaliana*, based on the microarray data of Affymetrix (ATH1), and several new reference genes were revealed [40]. This list of *Arabidopsis* reference genes was successfully employed to search for reference genes by sequence homology in unrelated species such as *Vitis vinifera*[7]. This approach resulted in a strategy that is based on the parallel use of a series of control genes and calculation of normalization factors using statistical algorithms [8,11,41]. It is necessary to validate the expression stability of a candidate control gene in each experimental system prior to its use for normalization. In this regard, several free software applications such as geNorm [8], NormFinder [42] or qBase [43] are used in order to identify the best internal controls from a group of candidate normalization genes in a given set of biological samples.

To our knowledge, no investigations have been yet carried out for the identification of reference genes in table grape, one of the most important template fruit crops. In this work we used a data set obtained from a large RNA-Seq experiment of table grape segregants phenotypically and genetically diverse, belonging to a 'Ruby Seedless’ x 'Sultanina’ crossing, sampled at three phenotypic stages, anthesis, fruit-setting and berries of 6–8 mm diameter (the last one from plants treated or not with gibberellic acid). We focused the search of control genes evaluating the variability (or stability) in the expression of 19 genes selected from an initial set of 242 genes that showed a threshold stability level, comparing the four different developmental and physiological conditions. Two new reference genes, *VvAIG1* (AvrRpt2-induced gene) and *VvTCPB* (T-complex 1 beta-like protein) were validated by qPCR and geNorm techniques and are presented as new housekeeping genes for table grape.

## Results and discussion

### Identification of putative reference genes

**Figure 1:**
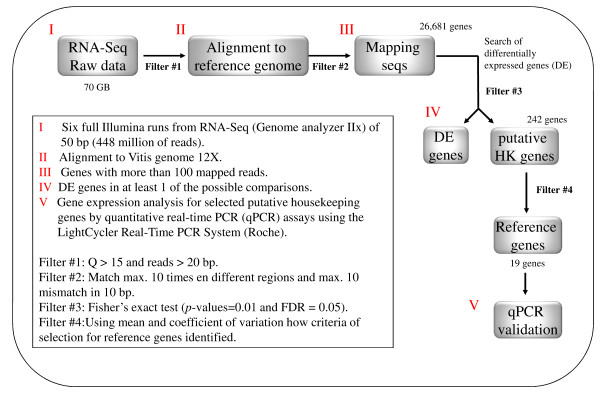
Bioinformatics pipeline used for the identification of the putative reference genes obtained through a high-throughput sequencing of cDNA (RNA-Seq).

Usually the search for reference genes in any plant species is based on the identification of orthologs of genes stably expressed in model plants, mainly from *Arabidopsis thaliana*[44,45]. In this case, we used our own information obtained from a massive sequencing assay done with 47 samples of the same species of interest, i.e., table grape (*Vitis vinifera* L.). This set of samples corresponds to 14 genotypes from which RNA was collected, combining different flower and berry developmental stages and treatments (see Methods section). Even when the main outcome of an RNA-Seq exercise is the identification of differentially expressed genes, in this case the same data set was used to search for putative reference genes, considered as that any gene that has a minimal expression level variation in every sample analyzed. Based on these criteria, a total of 242 candidate housekeeping genes were identified, using the bioinformatics workflow presented in Figure 1. These genes are involved in different biological processes (data not shown), such as synthesis, degradation, folding, defense, stress and catabolism of proteins and metabolites. As this number of genes is too large to evaluate each one respect of their transcriptional stability, we selected a subset according to statistical criteria described in the next section. With this purpose in mind, we ranked the list of 242 genes according to their coefficient of variation (*CV*), even when it was not observed a direct relation between *CV* and total reads (Additional file 1: Table S1).

### Selection of a sub-set of 19 candidate reference genes

Different approaches, such as Poisson distribution, quasi-Poisson distribution and negative binomial distribution, have been used to represent the statistical distribution of sequence data [46-48]. Under these three kinds of distribution, the mean of count reads is highly related to variance [47,48]. A summary of the three statistical parameters used in this work to characterize the NDE genes is shown in Table 1. The mean and the variance were high and positively related, while the average was not related to the *CV* (Table 2). Therefore, this last parameter, together with the mean, was used to select those NDE genes that behaved as housekeeping and had both a low variation coefficient and a high abundance along the 47 samples analyzed. The data (Table 1) showed a high estimated coefficient of variation (*CV* > 40%, with a range of ~25%). This variation probably could be given by an intrinsic variation within the biological sample (phenotype, phenological stages and gibberellic acid treatment) or by sampling error, because the sources of variation are considered during the selection of genes as differentially expressed by edgeR package [46]. Only a few genes (~8% of total NDE genes) had mean and *CV* values large enough to rule out sampling errors. This among-samples variation could be explained because the genotypic effect was not taken into account for the selection of NDE genes. In this study, each one of the 14 genotypes used could be differentially interacting with the other factors or conditions (phenotype, phenological stages and gibberellic acid treatment).

**Table 1:** **Descriptive statistics of mean (μ), variance (σ**^**2**^**) and coefficient of variation (*CV*) of the 242 non-differentially expressed genes**

	**Min.**	***q***_**1**_	**Median**	***q***_**3**_	**Max.**
*μ*	331.7	680.2	820.7	1129	3682
*σ*^*2*^	41300	120000	189400	326900	3636000
*CV*	0.444	0.492	0.515	0.544	0.648

Min.: minimum value; q1: first quartile; q3: third quartile; Max.: maximum value.

**Table 2:** Relationship among statistical parameters of read counts of non-differentially expressed genes (Pearson’s correlation coefficient)

	***μ***	***σ***^***2***^
*σ*^*2*^	0.951***	
*CV*	0.029 *n.s*.	0.126***

Significance codes: p value <0.001 '***’0.001-0.01 '**’0.01-0.05 '*’> 0.05 n.s. (non-significant); *μ* mean; *σ2* variance; *CV* coefficient of variation.

Because of the difficulties to find genes that possess simultaneously both a high expression level (number of reads) and a low *CV*, we used the coefficient of variation, which is not related to the mean and it is easier to interpret (Table 2). This parameter has been previously used in other experiments [49,50]. The threshold estimated by the simulation for *CV* and *μ* are listed in Table 3. According to this, as the threshold became more stringent, fewer genes were found that satisfied both criteria of selection: *CV* < percentile threshold and *μ* > percentile threshold (Table 3). Only 19 of the 242 NDE genes satisfied both criteria at 97.5% and 2.5% for *μ* and *CV*, respectively (Table 4).

**Table 3:** **Threshold used as criteria of selection based on the distribution of coefficient of variation (*CV*) and of mean (μ) of the 10,000 simulated genes**

***Percentile***	***Threshold***	***n***
*CV 5%*	0.536	34
*μ* 95%	1161.3	
*CV* 2.5%	0.513	19
*μ* 97.5%	1208.2	
*CV* 1%	0.487	4
*μ* 99%	1260.5	
*CV* 0.1%	0.43	0
*μ* 99.9%	1369.3	

n: Number of NDE genes that satisfied both criteria of selection: CV < percentile threshold and μ > percentile threshold.

**Table 4:** **Candidate reference gene ranking according to their** ***CV***

**Genoscope ID**	**Total reads**	**Mean**^**¥**^	**SD**	***CV***^**^**^	**CHR**	**Product**
GSVIVG01036166001*	80103	1704	791	0,46	chr6	Vacuolar protein sorting-associated protein 4
GSVIVG01013003001*	57177	1217	571	0,47	chr2	26S proteasome non-ATPase regulatory subunit 13
GSVIVG01027659001*	63133	1343	635	0,47	chr15	Unkown protein function
GSVIVG01025947001*	64396	1370	657	0,48	chr18	Protein AIG1
GSVIVG01035814001*	79018	1681	818	0,49	chr4	Unkown protein function
GSVIVG01038268001*	162669	3461	1689	0,49	chr5	Rab GDP dissociation inhibitor alpha
GSVIVG01008708001*	90480	1925	941	0,49	chr18	T-complex protein 1 subunit beta
GSVIVG01028520001*	96994	2064	1009	0,49	chr7	26S protease regulatory subunit 4 homolog
GSVIVG01012792001^ǂ^	56443	1201	588	0,49	chr18	Putative peptidase
GSVIVG01031067001	67287	1432	705	0,49	chr14	T-complex protein 1 subunit zeta
GSVIVG01033771001	83853	1784	883	0,49	chr8	Splicing factor U2af small subunit A
GSVIVG01033172001	78058	1661	822	0,50	chr4	Serine/Arginine-rich splicing factor 7
GSVIVG01016731001	69545	1480	734	0,50	chr9	Proteasome subunit alpha type-6
GSVIVG01028854001	82587	1757	875	0,50	chr16	40S ribosomal protein S10-1
GSVIVG01033442001*	63350	1348	673	0,50	chr8	Carbon catabolite repressor protein 4 homolog 2
GSVIVG01037814001*	70685	1504	754	0,50	chr3	Unkown protein function
GSVIVG01015062001^ǂ^	59049	1256	637	0,51	chr11	Aldehyde dehydrogenase family 7 member A1
GSVIVG01030215001*	155807	3315	1682	0,51	chr8	Proactivator polypeptide-like 1
GSVIVG01016593001*	101022	2149	1091	0,51	chr13	Actin-depolymerizing factor 2

*SD* standard deviation; *CV* coefficient of variation; *CHR* chromosome location for each gene.

*Genes studied in this work; ^ǂ^genes that showed double amplicon.

^¥^Threshold mean 1208.2 (percentile 97.5%).

^^^Threshold coefficient of variation 0.513 (percentile 2.5%).

### Primers design and analysis of the variability from threshold cycles value

Primer pairs for qPCR were designed and subsequently evaluated on table grape cDNA. For 17 out of the 19 primer pairs designed, a single amplicon was observed by electrophoretic separation; each amplicon was sequenced to confirm the primer specificity. The primers for *VvADH7* and *VvSLP* had to be excluded from the study as they produced two amplicons under the tested PCR conditions. All primers were designed with the following criteria: 20–24 bp length, GC content between 50% and 65%, product size in the range of 91–268 bp and melting temperature between 60–64°C (Table 5). Melting curve analyses of the 17 genes showed a single peak in each case, confirming that the primers amplified a single product (data not shown). Except for *VvUNP3* (129%) and *VvADF2* (114%), all PCRs displayed amplification efficiencies between 83% and 110% (Additional file 2: Table S2).

**Table 5:** List of primers designed for the 19 candidate reference genes considered in this study

**Genoscope ID**	**Gene abbreviation**	**GenBank accession**	**Primer sequence (5′-3′)**	**Product size (bp)**	**TM (°C)**
GSVIVT01038268001	*VvRABI*	XM_002280570	F: GCAAGGCTCAGTGCTGTTTA	217	60
			R: TTGGGATTGGGTGGCTCATA		
GSVIVT01030215001	*VvPP1*	XM_002268545	F: GAGCCAGGAATCCACAAAGAC	166	62
			R: AGAACCGACCAAACCCAAACT		
GSVIVT01016593001	*VvADF2*	XM_002284004	F: GGCCTTTGTCGCTGTTTCCT	268	60
			R: AGTGGGCTCACCAACCTTTT		
GSVIVT01028520001	*VvPR26S*	XM_002263298	F: GAGCAAGTTGAAGCCGCAGGAG	138	62
			R: CCCACGGACGACGACACGAT		
GSVIVT01008708001	*VvTCPB*	XM_002285876	F: AGACAGTGATTGACAGCCGAGTT	238	64
			R: ATCCCTGCGTGGCTTTCTTCC		
GSVIVT01033771001	*VvSFU2*	XM_002277409	F: CCCCACCCTCCTCCTTTCCAAC	192	64
			R: TGGTCAGCCAAATTGTCACAGA		
GSVIVT01028854001	*VvPR40*	XM_002273250	F: GATTGTGCCTGCCACCTTGA	257	62
			R: AACCTCCACCTCCTCGTCCA		
GSVIVT01036166001	*VvSAP4*	XM_002262726	F: AGCCTAATGTGAAGTGGAGC	179	60
			R: AACAGCCTTGGCTAGGTATG		
GSVIVT01035814001	*VvUNP2*	XM_002284964	F: AGATACAGAGGCAGGAGAAGT	214	64
			R: AGAATTGGGAATCCAGTGAGG		
GSVIVT01033172001	*VvSF7*	XM_002272621	F: GAGCGAGAACTTGAAGATGAG	258	62
			R: CAAACGGCATTCACGGGCAAA		
GSVIVT01037814001	*VvUNP3*	FQ387200	F: ACGCTCCTCAGTACGGTCAG	91	60
			R: AGAGCAGCCAAACATCCTTC		
GSVIVT01016731001	*VvPSA*	XM_002271893	F: ATGGACCTCGCCTCTTCAAAT	262	62
			R: TCCTCGGTGGACAACACTCTG		
GSVIVT01031067001	*VvTCPZ*	XM_002283474	F: CTTATGAAACAATCAGAACGCTAC	140	62
			R: TCAGGCTCATCACCCATTACCA		
GSVIVT01025947001	*VvAIG1*	XM_002281960	F: GAAGATTATTTGGGCCGTGAG	108	62
			R: CTTCTTGGCTTCATCCTTGGT		
GSVIVT01033442001	*VvCCRP*	XM_002280954	F: TTGGTTTGAAGTTGGACGCTCTA	173	64
			R: AGTGACGAGGAGTAGGTGAGG		
GSVIVT01027659001	*VvUNP*	XM_002280576	F: TCGGACCTTCGGATTAGCAT	227	60
			R: CACTCCAGTGGGTAGCATAG		
GSVIVT01015062001	*VvADH7*	XM_002278057	F: TCCGGCGAATCCTGGATGTTA	104	64
			R: CCGTCACCACCGCAATCCTCT		
GSVIVT01013003001	*VvPRN26S*	XM_003631440	F: GAAGCTCTGGCACCAACTCACT	158	64
			R: ACTGCCTAGAAACTATGACAGCAA		
GSVIVT01012792001	*VvSLP*	FQ388031	F: GCCGTCCACATCATTTACACT	108	62
			R: AGCCTTCTTGGCAGCCTCCTC		

*F* forward primer. *R* reverse primer. *bp* base pairs. *TM* melting temperatura given in °C.

**Figure 2:**
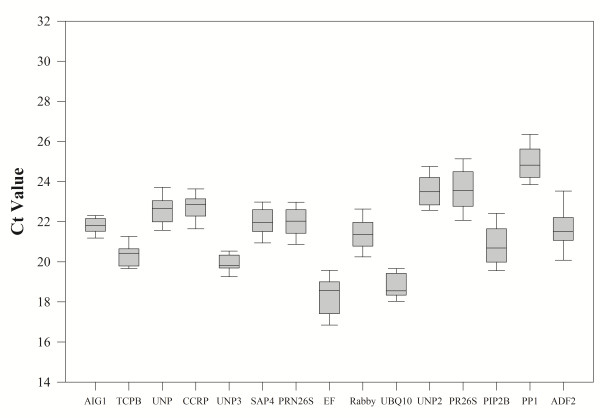
**Variability of threshold cycles (Ct) value in each reference gene among all tested samples.** A line across the box indicates the median. The box indicates the 25th and 75th percentiles. Whiskers represent the maximum and minimum values.

As a first approach we compared the different expression levels of the reference genes over all the 47 samples using the absolute Ct value. Analysis of the raw expression levels across all samples detected some variation among reference genes. The results (Additional file 2: Table S2 and Figure 2) revealed that all genes presented median ct values between 18.5 and 24.8 and the CV was < 7% for all the reference genes (Additional file 2: Table S2), among which *VvAIG1* and *VvTCPB* presented the lowest *CV*s, 3.6 and 3.9 respectively.

### Expression analysis of reference genes for qPCR

**Figure 3:**
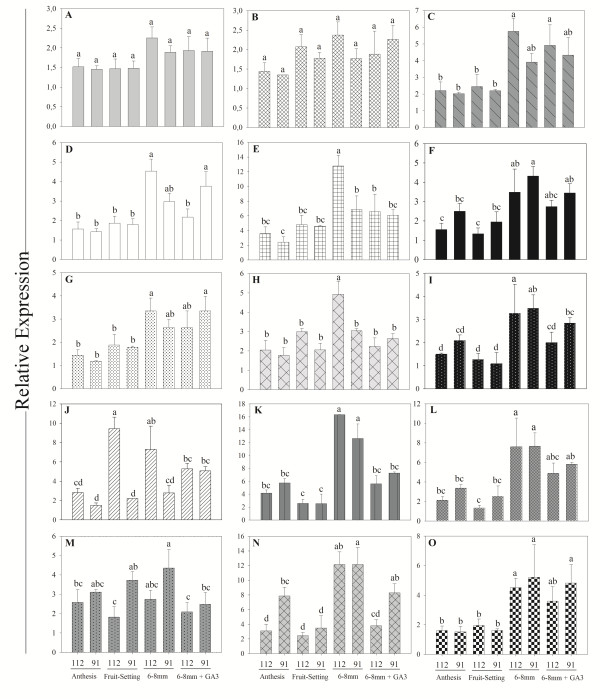
**qPCR expression values for candidate reference genes in grapevine samples.** Two segregants from the Ruby x Sultanina crossing (112 and 19) in three phenological stages (anthesis, fruit-setting and 6–8 mm berries) treated or not with gibberellic acid (GA3) were used. These segregants represent extreme phenotypes for berry size. For relative expression the genes were normalized with the lowest expression gene. **A**, AIG1 (*VvAIG1*); **B**, T-complex protein 1 subunit beta (*VvTCPB*); **C**, vacuolar sorting-associated protein 4 (*VvSAP4*); **D**, 26S proteasome non-ATPase regulatory subunit 13 (*VvPRN26S*); **E**, carbon catabolite repressor protein 4 homolog 2 (*VvCCRP*); **F**, unkown protein function (*VvUNP2*); **G**, unkown protein function (*VvUNP*); **H**, unkown protein function (*VvUNP3*); **I**, Rab GDP dissociation inhibitor alpha (*VvRABI*); **J**, proactivator polypeptide-like 1 (*VvPP1*); **K**, acting-depolymerizing factor 2 (*VvADF2*); **L**, 26S protease regulatory subunit 4 homolog (*VvPR26S*). Other putative housekeeping genes reported and used in many works are the following: **M**, polyubiquitin (*VvUBQ10*, GenBank acc CB977307); **N**, plasma membrane intrinsic protein 2B (*VvPIP2B*, GenBank acc EC969993); and **O**, elongation factor 1-alpha (*VvEF*1-α, GenBank acc CB977561). Bars in the graphs correspond to standard error (SE) from three biological samples, assayed in duplicate. Different letters represent significant differences a t *P* < 0.05 by LSD test.

Using quantitative Real-Time PCR we studied the expression of 12 out of 19 candidate reference genes in cDNA samples of table grape genotypes from different phenological stages. Most of the genes showed a similar expression pattern considering the different samples under study, e.g., lower expression at anthesis and fruit-setting stages and slightly higher expression in the 6–8 mm berry size stage (Figure 3, C-L). Other genes such as *VvAIG1* and *VvTCPB* did not show significant differences in their expression along the different phenological stages and in the different samples (segregants) studied (Figure 3, A and B). As a control, we included three genes studied by Reid et al. [7], *VvUBQ10*, *VvPIP2B* and *VvEF1-α*, which presented an expression profile similar to the set of putative reference genes, with appreciable differences between phenological stages (Figure 3, M-O). Interestingly, this set of three control genes, commonly used in gene expression studies in grapevine exhibited very “unstable”, non-uniform or too-low expression levels, and so they were not included in the list of 242 genes initially selected, and consequently they are not recommendable to be used as reference genes in table grapes.

### Validation of candidate reference genes

**Figure 4:**
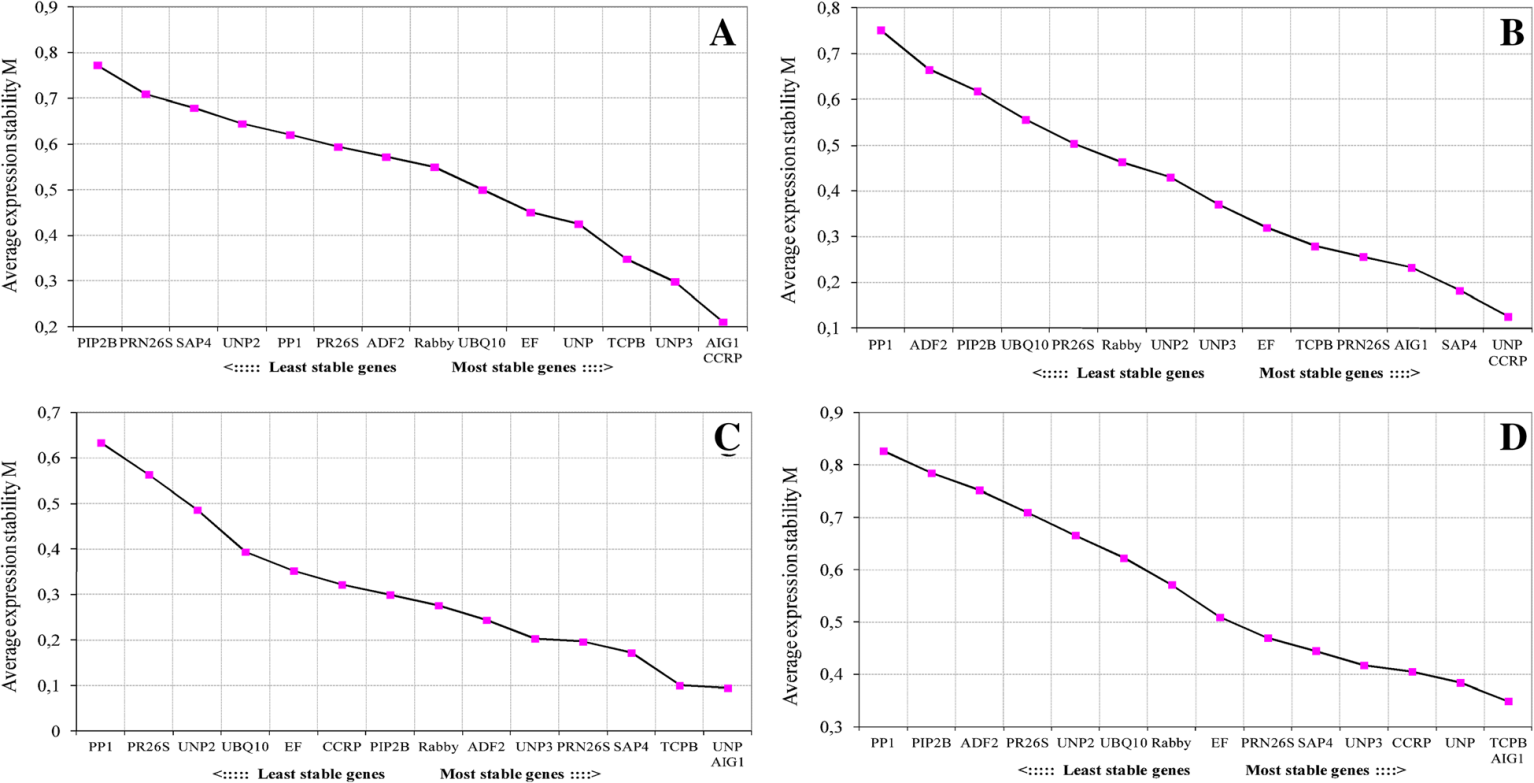
**Expression stability values (M) and ranking of 14 candidate housekeeping genes as calculated by geNORM algorithm.** Average expression stability value (M) of the candidate genes was measured during stepwise exclusion of the least stable candidate genes. Genes with the lowest M values have the most stable expression. Twenty-four cDNAs corresponding to different phenological stages were used: **A**, anthesis; **B**, fruit-setting; **C**, 6–8 mm berries; and **D** represents all the phenological stages.

**Figure 5:**
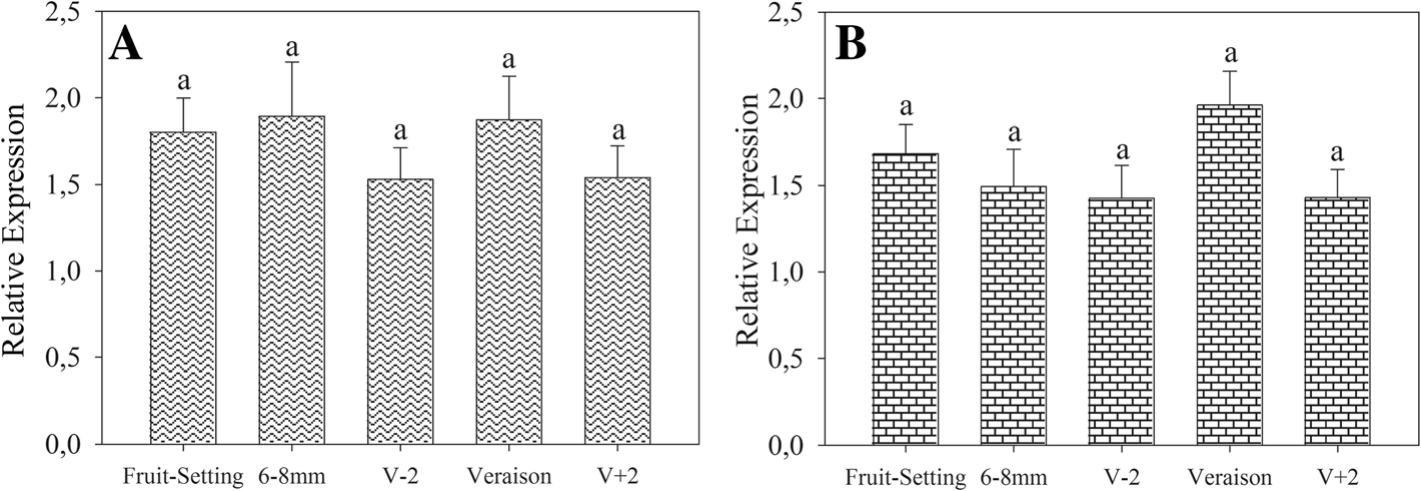
**Validation by qPCR of two putative reference genes in cDNA from 'Sultanina’ samples.** We selected 20 samples from five different phenological stages: before (fruit-setting, 6–8 mm berry size and V-2) and after (V + 2) veraison (V). **A**, AIG1 (*VvAIG1*) and **B**, T-complex protein 1 subunit beta (*VvTCPB*). Bars in the graphs correspond to standard error (SE) from four biological samples, Different letters represent significant differences at *P* < 0.05 by LSD test.

For the validation of *VvAIG1* and *VvTCPB* as reference genes, we studied their expression profile also in more advanced phenological stages (pre- veraison and post- veraison), using cv. Sultanina as a model table grape genotype. Some authors as Gamm et al. [34] and Artico et al. [23] among others, recommend that the ideal reference genes should be expressed at a constant level throughout the plant tissues, developmental stages or physiological conditions, and not be influenced by exogenous treatments but no one gene has such a stable expression under every experimental condition, as numerous studies reported that expression of housekeeping genes can also vary considerably under particular experimental conditions as it is observed in the Figure 4. *VvAIG1* and *VvTCPB* genes neither presented significant differences in their expression at the growing stages evaluated (Figures 5A and 5B, respectively). Similar results for these two genes were observed in cvs. Red Globe, Crimson seedless and Muscat of Alexandria, a set of genotypes representing at some extent the genetic diversity of table grapes [51]. In addition, we evaluated the performance of these two reference genes in leaves with similar results as berries (data not shown).

To complement this, we used geNorm algorithm to determine the most stable reference genes assuming that two ideal reference genes should not vary in comparison with each other in the different tested conditions. This algorithm calculates the average pair wise variation of a given candidate reference genes set with all other genes under evaluation and assigns a measure of its expression stability (M), based on which a ranking of candidate reference genes is produced [8]. The geNorm software has been cited for many authors in relation to the identification or behavior of reference genes; this is because of its easiness, robustness, reliability and convenience of use, and so it is currently included in qRT-PCR analyses in animals, yeasts, bacteria but rarely in plants [52]. Our results based on geNorm were consistent with this couple of genes being very stable regarding gene expression in the analyzed samples.

For anthesis, the two most stable genes were *VvAIG1* and *VvCCRP* (Figure 4A); in the case of fruit-setting these were *VvUNP* and *VvCCRP* (Figure 4B); and for 6–8 mm berries, the most stable genes were *VvUNP* and *VvAIG1* (Figure 4C). Other genes considered in this work (*EF*, *PP2A* and *UBQ10*) were studied in other species of plants such as soybean [53] and *Gossypium hirsutum*[23], showing a high variability in their expression profile depending of the physiological condition, tissues and genotypes.

In summary, the most stable reference genes for all samples studied (different genotypes evaluated at different phenological stages) were *VvTCPB* and *VvAIG1* (Figure 4D). These results demonstrate that our approach allowed us to obtain a set of genes that could be used as reference genes in qPCR experiments; this is similar to the result obtained by Coito et al. [40], where they proved the accuracy of choosing a combination of grapevine reference genes for qPCR, but in that case through a microarray analysis.

## Conclusions

This work is the first study that shows that a data set derived from a massive RNA sequencing for several individuals and phenotypic conditions can be used for the identification of housekeeping genes in a non-model plant species such as grapevine. The genes *VvTCPB* and *VvAIG1*, never cited before as possible reference genes in this or other woody species were the most stable genes in all samples studied. Then, these genes are proposed as reference genes to be used in qPCR assays in table grape berries at different developmental stages and physiological conditions.

## Methods

### Plant material

Twelve table grape segregants belonging to a 'Ruby seedless’ x 'Sultanina’ crossing of contrasting and extreme phenotypes respect of seed content and berry size plus both parents were used in the RNA-Seq experiments (Muñoz et al., manuscript in preparation). For RNA-Seq analyses, a number of whole berries from each condition (for a list of samples, phenological stages, etc., see Additional file 3: Table S3) was frozen in liquid nitrogen, homogenized and their RNA was sequenced after converted to cDNA, obtaining ca. 500 million reads from 47 sequenced samples.

For the qPCR validation of the 19 candidate reference genes, two genotypes from the same crossing collected at three phenological stages (anthesis, fruit-setting and 6–8 mm berries, treated or not with gibberellic acid) were used. We also included samples of 'Sultanina’ collected at more advanced phenological stages (pre-veraison and post-veraison). The vines, established at La Platina Experimental Station of the 'Instituto de Investigaciones Agropecuarias’, located in Santiago, Chile, were maintained under a standard management program for watering, fertilization, pests and diseases control and pruning. After harvest, every sample was immediately frozen in liquid nitrogen and stored at -80°C until use.

### Public data used

The reference grape genome (12X) and the gene annotation were downloaded from the GENOSCOPE database (http://www.genoscope.cns.fr/externe/GenomeBrowser/Vitis/). The reference genome contains a total of 26,346 annotated transcripts with an average size of 1,137 base pairs.

### Identification of candidate reference genes

To build the RNA-Seq data-base, a total of 491 million reads were generated in a Genome Analyzer II, from Illumina (IGA, Udine, Italy). After the quality trimming, 477 million reads were kept, and 91% of them were located in the reference grape genome by TOPHAT [54] program. The differential expression test on seventy comparisons was implemented in the edgeR [46] software. Then, using in-house development scripts, we searched for genes that were classified as non-differentially expressed, and presented at least 100 reads in each sample/condition and a low variation index among conditions. Finally, all these steps were executed as a bash pipeline (Figure 1).

### Derivation of the statistical test for the selection of reference genes

As a first approximation to identify the reference genes, it was used as criteria the mean of read counts and the coefficient of variation (*CV* = standard deviation/mean) among the 47 different conditions for each of the 242 non-differentially expressed genes (NDE). The relationship between these two criteria was analyzed by Pearson’s correlation coefficient (*r*) using R 2.15.0 [55]. The *CV* has been previously used for this purpose in cereal crops [49,50]. In order to find those genes having both a high number of reads and a low variation coefficient among samples from different phenological stages and conditions, pseudo data sets were simulated by resampling of the original data. The purpose was that the stability (low *CV*) and level of expression (high mean values of read counts) were due to features of the gene and not to random or experimental error. The procedure was performed as follows: for each original gene we calculated the mean and the *CV* of the read counts among the different conditions. Then, a pseudo set of data was simulated representing a pseudo NDE gene under the 47 conditions. To represent this gene, 47 read counts were sampled at random from the original data matrix (247 × 47 observations) and then both the mean and *CV* were calculated for this pseudo NDE gene. Thus, 10,000 pseudo NDE genes were simulated. Then the 10,000 pseudo-values of the mean and *CV* were sorted from the lowest to the highest values. The highest 9,750-th value (percentile: 97.5%) and the lowest 250-th value (percentile: 2.5%) of mean and *CV*, respectively, were used as thresholds of selection. Finally, only those genes that had both a mean of read counts above and a *CV* below the corresponding thresholds were selected. This algorithm was programmed using R 2.15.0 [55].

### RNA isolation and cDNA synthesis

Total RNA was isolated from 3–4 g of frozen tissue using the modified hot borate method [56]. The quantity and quality of the RNA were assessed by measuring the A_260/280_ ratio and by electrophoresis on a 1.2% formaldehyde-agarose gel. First strands of cDNA were obtained by reverse transcription reactions with 2 ug of total RNA as template, using MMLV-RT reverse transcriptase (Promega, Madison, WI) and oligo dT primers according to standard procedures. The concentration of cDNA was assessed by measuring the absorbance at 260 nm, finally diluting each cDNA to 50 ng/ul prior to use in qPCR. Quality and quantity of cDNA was also determined by using a Bioanalyzer (Agilent Technologies, Santa Clara, CA), with equivalent results.

### Primer design

Gene-specific primers were designed using Primer Premier 5.0 software (Premier Biosoft International, Palo Alto, CA) and synthesized by Alpha DNA (Montreal, Quebec, Canada). The nucleotide sequences were obtained from a private data-base maintained at http://vitisdb.cmm.uchile.cl/. In addition, three genes encoding a polyubiquitin (UBQ10), plasma membrane intrinsic protein 2B (PIP2B) and elongation factor 1-alpha (EF-1α) and their respective pairs of primers were selected from previously published reports [28] and evaluated as a way of comparison. Accession numbers, primer sequences, expected size of amplicons and melting temperature are provided in Table 5.

### Quantitative real-time PCR assays (qPCR)

Each transcript abundance was analyzed by real-time PCR with the LightCycler Real-Time PCR System (Roche Diagnostics, Mannheim, Germany), using SYBR Green™ as a fluorescent dye to measure the amplified DNA products derived from RNA. Three biological samples in duplicate of quantitative PCR experiments were performed for each sample as described in García-Rojas et al. [57]. Briefly, the amplification reaction was carried out in a total volume of 20 μl containing 1 pmol of each primer, 5 mM MgCl_2_, 1 ml LightCycler™ DNA Master SYBR® Green I (Roche Diagnostics) and 100 ng of each cDNA analyzed. The thermal cycle conditions were: denaturation at 95°C for 10 min, followed by 35 three-step cycles of template denaturation at 95°C with a 2 s hold, primer annealing at 60–65°C for 15 s and extension at 72°C for 25 s. Fluorescence data was collected after each extension step. Melting curve analyses were performed and checked for single peaks, and the amplification product sizes were confirmed in an agarose gel to ensure the absence of non-specific PCR products. Fluorescence was analyzed using LightCycler™ Analysis Software (Roche Diagnostics). The crossing point for each reaction was determined using the Second Derivative Maximum algorithm and manual baseline adjustment.

### Determination of reference gene expression stability

Expression levels of each one of the 19 candidate reference genes in all samples were determined by assessing the number of threshold cycles (Ct) needed for the amplification related fluorescence to reach a specific threshold level detection. Ct values were transformed to quantities using a standard curve which is a requirement for using geNorm. To manage the large number of calculations generated, we used a Visual Basic Application (VBA) for Microsoft Excel that automatically calculates the gene-stability value M for every control gene in a given set of samples [8].

### Statistical analysis for qPCR

Data from qPCR was subjected to statistical analysis of variance, and means were separated by LSD test at 5% level of significance using Statgraphics Plus 5 (Manugistics Inc., Rockville, MD).

The RNA-Seq data used in this study is available at the NCBI’s Sequence Read Achieve (http://www.ncbi.nlm.nih.gov/sra) with the SRA Study accession number SRX366617.

## Competing interests

The authors declare that they have no competing interests.

## Authors’ contributions

MGA Selection of the criteria to retrieve the reference genes from the microarray, project CoI, writing the manuscript; MGR experimental lab work, application of the softwares and statistical methods, writing the manuscript; AD statistical and bioinformatics analysis; JC experimental field work, statistical analysis; AM project CoI, revision of the manuscript; AO project CoI, revision of the manuscript; PH design of the study, project leader, revision of the manuscript. All authors contributed to the design of the experiments, and read and approved the final manuscript.

## Supplementary Material

Additional file 1: Table S1 List of the 242 candidate reference genes ranked according to their *CV* values. Click here for file

Additional file 2: Table S2 Determination of threshold values (Ct) obtained from qPCR analyses. Click here for file

Additional file 3: Table S3 List of the samples and conditions used for the RNA-Seq experiments. Click here for file

## References

[B1] Bustin SA Absolute quantification of mRNA using real-time reverse transcription polymerase chain reaction assays J Mol Endocrinol 2000 14 169 193 10.1677/jme.0.0250169 11013345

[B2] Chuaqui RF Bonner RF Best CJ Gillespie JW Flaig MJ Hewitt SM Phillips JL Krizman DB Tangrea MA Ahram M Linehan WM Knezevic V Emmert-Buck MR Post-analysis follow-up and validation of microarray experiments Nat Genet 2002 14 509 514 10.1038/ng1034 12454646

[B3] Canales RD Luo Y Willey JC Austermiller B Barbacioru CC Boysen C Hunkapiller K Jensen RV Evaluation of DNA microarray results with quantitative gene expression platforms Nat Biotech 2006 14 1115 1122 10.1038/nbt1236 16964225

[B4] Hao QN Zhou XA Sha AH Wang C Zhou R Chen SL Identification of genes associated with nitrogen-use efficiency by genome-wide transcriptional analysis of two soybean genotypes BMC Genomics 2011 14 525 10.1186/1471-2164-12-525 22029603 PMC3210170

[B5] de Jonge H Fehrmann R de Bont E Hofstra R Gerbens F Kamps W de Vries E van der Zee A te Meerman G ter Elst A Evidence based selection of housekeeping genes PLoS One 2007 14 e898 10.1371/journal.pone.0000898 17878933 PMC1976390

[B6] Dheda K Huggett JF Chang JS Kim LU Bustin SA Johnson MA Rook GAW Zumla A The implications of using an inappropriate reference gene for real-time reverse transcription PCR data normalization Anal Biochem 2005 14 141 143 10.1016/j.ab.2005.05.022 16054107

[B7] Reid K Olsson N Schlosser J Peng F Lund S An optimized grapevine RNA isolation procedure and statistical determination of reference genes for real-time RT-PCR during berry development BMC Plant Biol 2006 14 27 37 10.1186/1471-2229-6-27 17105665 PMC1654153

[B8] Vandesompele J De Preter K Pattyn F Poppe B Van Roy N De Paepe A Speleman F Accurate normalization of real-time quantitative RT-PCR data by geometric averaging of multiple internal control genes Genome Biol 2002 14 1 11 10.1186/gb-2002-3-7-research0034 PMC126239 12184808

[B9] Huggett J Dheda K Bustin SA Dorak MT Normalization Real-time PCR 2006 New York: BIOS Advanced Methods 83 91

[B10] Exposito-Rodriguez M Borges A Borges-Perez A Perez J Selection of internal control genes for quantitative real-time RT-PCR studies during tomato development process BMC Plant Biol 2008 14 131 10.1186/1471-2229-8-131 19102748 PMC2629474

[B11] Thellin O Zorzi W Lakaye B De Borman B Coumans B Hennen G Grisar T Igout A Heinen E Housekeeping genes as internal standards: use and limits J Biotech 1999 14 291 295 10.1016/S0168-1656(99)00163-7 10617337

[B12] Libus J Štorchová H Quantification of cDNA generated by reverse transcription of total RNA provides a simple alternative tool for quantitative RT-PCR normalization Biotechniques 2006 14 156 164 10.2144/000112232 16925017

[B13] Nolan T Hands RE Bustin SA Quantification of mRNA using real-time RT-PCR Nat Protoc 2006 14 1559 1582 10.1038/nprot.2006.236 17406449

[B14] VanGuilder HD Vrana KE Freeman WM Twenty-five years of quantitative PCR for gene expression analysis Biotechniques 2008 14 619 626 18474036 10.2144/000112776

[B15] Suzuki T Higgins PJ Crawford DR Control selection for RNA quantitation Biotechniques 2000 14 332 337 10948434 10.2144/00292rv02

[B16] Foss DL Baarsch MJ Murtaugh MP Regulation of hypoxanthine phosphoribosyltransferase, glyceraldehyde-3-phosphate dehydrogenase and beta-actin mRNA expression in porcine immune cells and tissues Animal Biotech 1998 14 67 78 10.1080/10495399809525893 9676236

[B17] Schmittgen T Zakrajsek B Effect of experimental treatment on housekeeping gene expression: validation by real-time, quantitative RT-PCR J Biochem Biophys Meth 2000 14 69 81 10.1016/S0165-022X(00)00129-9 11086195

[B18] Warrington JA Nair A Mahadevappa M Tsyganskaya M Comparison of human adult and fetal expression and identification of 535 housekeeping/maintenance genes Physiol Genom 2000 14 143 147 10.1152/physiolgenomics.2000.2.3.143 11015593

[B19] Selvey S Thompson EW Matthaei K Lea RA Irving MG Griffiths LR Beta-actin an unsuitable internal control for RT-PCR Mol Cell Probes 2001 14 307 311 10.1006/mcpr.2001.0376 11735303

[B20] Lee PD Sladek R Greenwood CM Hudson TJ Control genes and variability: absence of ubiquitous reference transcripts in diverse mammalian expression studies Genome Res 2002 14 292 297 10.1101/gr.217802 11827948 PMC155273

[B21] Glare EM Divjak M Bailey MJ Walters EH β-Actin and GAPDH housekeeping gene expression in asthmatic airways is variable and not suitable for normalizing mRNA levels Thorax 2002 14 765 770 10.1136/thorax.57.9.765 12200519 PMC1746418

[B22] Czechowsky T Stitt M Altmann T Udvardi K Scheible WR Genome-wide identification and testing of superior reference genes for transcript normalization in *Arabidopsis* Plant Physiol 2005 14 5 17 10.1104/pp.105.063743 16166256 PMC1203353

[B23] Artico S Nardelli SM Brilhante O Grossi-de-Sa MF Alves-Ferreira M Identification and evaluation of new reference genes in *Gossypium hirsutum* for accurate normalisation or real-time quantitative RT-PCR data BMC Plant Biol 2010 14 49 10.1186/1471-2229-10-49 20302670 PMC2923523

[B24] Abbal P Pradal M Muniz L Sauvage FX Chatelet P Ueda T Tesniere C Molecular characterization and expression analysis of the Rab GTPase family in *Vitis vinifera* reveal the specific expression of a VvRabA protein J Exp Bot 2008 14 2403 2416 10.1093/jxb/ern132 18499650

[B25] Muñoz-Robredo P Gudenschwager O Chervin C Campos-Vargas R González-Agüero M Defilippi BG Study on differential expression of 1-aminocyclopropane-1-carboxylic acid oxidase genes in table grape cv. Thompson seedless Postharvest Biol Tec 2013 14 163 169

[B26] Fung RW Gonzalo M Fekete C Kovacs LG He Y Marsh E McIntyre LM Schachtman DP Qiu W Powdery mildew induces defense-oriented reprogramming of the transcriptome in a susceptible but not in a resistant grapevine Plant Physiol 2008 14 236 249 17993546 10.1104/pp.107.108712 PMC2230561

[B27] Ruan W Lai M Actin, a reliable marker of internal control Clin Chim Acta 2007 14 1 5 10.1016/j.cca.2007.07.003 17698053

[B28] Lund ST Peng FY Nayar T Reid KE Schlosser J Gene expression analyses in individual grape (*Vitis vinifera* L.) berries during ripening initiation reveal that pigmentation intensity is a valid indicator of developmental staging within the cluster Plant Mol Biol 2008 14 301 315 10.1007/s11103-008-9371-z 18642093

[B29] Olsen KM Hehn A Jugde H Slimestad R Larbat R Bourgaud F Lillo C Identification and characterization of CYP75A31, a new Flavonoid 3′5′-hydroxylase, isolated from *Solanum lycopersicum* BMC Plant Biol 2010 14 21 10.1186/1471-2229-10-21 20128892 PMC2825239

[B30] Bas A Forsberg G Hammarstrom S Hammarstrom ML Utility of the housekeeping genes 18S rRNA, beta-actin and glyceraldehyde- 3-phosphate-dehydrogenase for normalization in real-time quantitative reverse transcriptase-polymerase chain reaction analysis of gene expression in human T lymphocytes Scan J Immunol 2004 14 566 573 10.1111/j.0300-9475.2004.01440.x 15182252

[B31] Wang X Liu W Chen X Tang C Dong Y Ma J Huang X Wei G Han Q Huang L Kang Z Differential gene expression in incompatible interaction between wheat and stripe rust fungus revealed by cDNA-AFLP and comparison to compatible interaction BMC Plant Biol 2010 14 9 10.1186/1471-2229-10-9 20067621 PMC2817678

[B32] Xue JL Salem TZ Turney CM Cheng XW Strategy of the use of 28S rRNA as a housekeeping gene in real-time quantitative PCR analysis of gene transcription in insect cells infected by viruses J Virol Methods 2010 14 210 215 10.1016/j.jviromet.2009.09.019 19781576

[B33] Thellin O El Moualij B Heinen E Zorzi W A decade of improvements in quantification of gene expression and internal standard selection Biotechnol Adv 2009 14 323 333 10.1016/j.biotechadv.2009.01.010 19472509

[B34] Gamm M Héloir MC Kelloniemi J Poinssot B Wendehenne D Adrian M Identification of reference genes suitable for qRTPCR in grapevine and application for the study of the expression of genes involved in pterostilbene synthesis Mol Genet Genomics 2011 14 273 285 10.1007/s00438-011-0607-2 21340517

[B35] Jain M Nijhawan A Tyagi AK Khurana JP Validation of housekeeping genes as internal control for studying gene expression in rice by quantitative real-time PCR Biochem Bioph Res Co 2006 14 646 651 10.1016/j.bbrc.2006.04.140 16690022

[B36] Jian B Liu B Bi Y Hou W Wu C Han T Validation of internal control for gene expression study in soybean by quantitative real-time PCR BMC Mol Biol 2008 14 59 10.1186/1471-2199-9-59 18573215 PMC2443375

[B37] Hong S Yang M Xiang F Park C Exploring valid reference genes for gene expression studies in *Brachypodium distachyon* by real-time PCR BMC Plant Biol 2008 14 112 10.1186/1471-2229-8-112 18992143 PMC2588586

[B38] De Boever S Vangestel C De Backer P Croubels S Sys S Identification and validation of housekeeping genes as internal control for gene expression in an intravenous LPS inflammation model in chickens Vet Immunol Immunop 2008 14 312 317 10.1016/j.vetimm.2007.12.002 18272235

[B39] Ransbotyn V Reusch T Housekeeping gene selection for quantitative real-time PCR assays in the seagrass *Zostera marina* subjected to heat stress Limnol Oceanogr-Meth 2006 14 367 373

[B40] Coito JL Rocheta M Carvalho L Amâncio S Microarray-based uncovering reference genes for quantitative real time PCR in grapevine under abiotic stress BMC Res Notes 2012 14 220 10.1186/1756-0500-5-220 22564373 PMC3837474

[B41] Bustin SA Quantification of mRNA using real-time reverse transcription PCR (RT-PCR): trends and problems J Mol Endocrinol 2002 14 23 39 10.1677/jme.0.0290023 12200227

[B42] Andersen CL Jensen JL Orntoft TF Normalization of real-time quantitative reverse transcription-PCR data: a model-based variance estimation approach to identify genes suited for normalization, applied to bladder and colon cancer data sets Cancer Res 2004 14 5245 5250 10.1158/0008-5472.CAN-04-0496 15289330

[B43] Hellemans J Mortier G De Paepe A Speleman F Vandesompele J qBase relative quantification framework and software for management and automated analysis of real-time quantitative PCR data Genome Biol 2007 14 R19 10.1186/gb-2007-8-2-r19 17291332 PMC1852402

[B44] Demidenko NV Logacheva MD Penin AA Selection and validation of reference genes for quantitative real-time PCR in buckwheat (*Fagopyrum esculentum*) based on transcriptome sequence data PLoS One 2011 14 e19434 10.1371/journal.pone.0019434 21589908 PMC3093374

[B45] Pellino M Sharbel TF Mau M Amiteye S Corral JM Selection of reference genes for quantitative real-time PCR expression studies of microdissected reproductive tissues in apomictic and sexual *Boechera* BMC Res Notes 2011 14 303 10.1186/1756-0500-4-303 21851639 PMC3171723

[B46] Robinson M McCarthy D Smyth G edgeR: a bioconductor package for differential expression analysis of digital gene expression data Bioinformatics 2010 14 139 140 10.1093/bioinformatics/btp616 19910308 PMC2796818

[B47] Kvam V Liu P Si Y A comparison of statistical methods for detecting differentially expressed genes from RNA-Seq data Am J Bot 2012 14 248 256 10.3732/ajb.1100340 22268221

[B48] McCarthy D Chen Y Smyth G Differential expression analysis of multifactor RNA-Seq experiments with respect to biological variation Nucl Acids Res 2012 14 4288 4297 10.1093/nar/gks042 22287627 PMC3378882

[B49] Wang Y Li X Mao Y Blaschek H Single-nucleotide resolution analysis of the transcriptome structure of *Clostridium beijerinckii* NCIMB 8052 using RNA-Seq BMC Genom 2011 14 479 10.1186/1471-2164-12-479 PMC3271303 21962126

[B50] Yang S Tu Z Cheung F Xu W Lamb J Jung H Vance C John W Gronwald J Using RNA-Seq for gene identification polymorphism detection and transcript profiling in two alfalfa genotypes with divergent cell wall composition in stems BMC Genom 2011 14 199 10.1186/1471-2164-12-199 PMC3112146 21504589

[B51] Aradhya KM Dangl GS Prins BH Boursiquot JM Walker AM Meredith CP Simon CJ Genetic structure and differentiation in cultivated grapes, *Vitis vinifera* L Gen Res 2003 14 179 192 10.1017/S0016672303006177 12929909

[B52] Guenin S Mauriat M Pelloux J Van Wuytswinkel O Bellini C Gutierrez L Normalization of qRT-PCR data: the necessity of adopting a systematic, experimental conditions-specific validation of references J Exp Bot 2009 14 487 493 10.1093/jxb/ern305 19264760

[B53] Hu R Fan C Li H Zhang Q Fu YF Evaluation of putative reference genes for gene expression normalization in soybean by quantitative real-time RT-PCR BMC Mol Biol 2009 14 93 10.1186/1471-2199-10-93 19785741 PMC2761916

[B54] Trapnell C Pachter L Salzberg SL TopHat: discovering splice junctions with RNA-Seq Bioinformatics 2009 14 1105 1111 10.1093/bioinformatics/btp120 19289445 PMC2672628

[B55] R Development Core Team R: a language and environment for statistical computing Vienna, Austria: R Foundation for Statistical Computing ISBN 3-900051-07-0, URL http://www.R-project.org/, 2012

[B56] Gudenschwager O González-Agüero M Defilippi BG A general method for high-quality RNA isolation from metabolite-rich fruits S Afr J Bot 2012 14 186 192

[B57] García-Rojas M Gudenschwager O Defilippi BG González-Agüero M Identification of genes possibly related to loss of quality in late-season 'Hass’ avocados in Chile Postharvest Biol Tec 2012 14 1 7

